# Talar OsteoPeriostic Grafting From the Iliac Crest (TOPIC): Prospective 2-Year Outcomes for Large Lateral Osteochondral Lesions of the Talus

**DOI:** 10.1177/10711007251329033

**Published:** 2025-04-27

**Authors:** Julian J. Hollander, Kaj S. Emanuel, Jari Dahmen, Gino M. M. J. Kerkhoffs, Sjoerd A. S. Stufkens

**Affiliations:** 1 Department of Orthopaedic Surgery and Sports Medicine, Amsterdam UMC, Location AMC, University of Amsterdam, Amsterdam, the Netherlands; 2 Amsterdam Movement Sciences, Programs Sports and Musculoskeletal Health, Amsterdam, the Netherlands; 3 Academic Center for Evidence based Sports medicine (ACES), Amsterdam UMC, Amsterdam, the Netherlands; 4 Amsterdam Collaboration for Health and Safety in Sports (ACHSS), International Olympic Committee (IOC) Research Center, Amsterdam UMC, Amsterdam, the Netherlands

**Keywords:** osteochondral lesion, talus, ankle, foot, TOPIC, osteochondral autograft, periosteal graft

## Abstract

**Background:**

The results of the Talar OsteoPeriostic grafting from the Iliac Crest (TOPIC) procedure for lateral osteochondral lesions of the talus (OLTs) are unknown. Therefore, the present prospective study aims to assess the numeric rating scale (NRS) of pain during walking at 2-year follow-up. Secondarily, the aim is to assess other clinical, radiologic, and safety outcomes.

**Methods:**

This is a single-center, nonrandomized prospective cohort study in which all press-fit lateral TOPIC patients for an OLT are included. Patients with a follow-up of at least 2 years without a concomitant osteochondral lesion of the tibial plafond were included. The primary outcome is the NRS of pain during walking. Secondary clinical outcomes included the NRS during rest and during stair climbing. Additionally, the Foot and Ankle Outcome Score (FAOS), the AOFAS ankle-hindfoot score, and the mental and physical component summary of the 36-Item Short Form Health Survey were recorded. Radiologic follow-up was performed using computed tomography (CT) scans.

**Results:**

After application of the inclusion and exclusion criteria, 7 patients were included in the present study. The median age at time of surgery was 31.1 years. The NRS of pain during walking improved from a median of 5 (4-7) preoperatively to 1 (0-1) at 2 years of follow-up (*P* = .02). All FAOS subscales improved significantly, except the FAOS symptoms subscale. Graft consolidation was observed in 100% of the patients and cysts were present in 5 of 6 patients. No complications occurred and no patients complained of donor site morbidity. No reoperations were performed.

**Conclusion:**

In the first 7 prospectively followed patients who underwent the TOPIC procedure for large osteochondral lesions of the lateral talar dome, an improvement of the NRS of pain during walking from median 5 preoperatively to 1 at 2-year follow-up was observed.

**Level of Evidence:** Level IV, therapeutic.

## Introduction

Osteochondral lesions of the talus (OLTs) are lesions of both the cartilage and underlying subchondral bone plate. These lesions have a profound impact on the quality of life of patients.^8^ Patients often complain of deep ankle pain during activity. To treat these patients, several options are available, both operatively and nonoperatively. No consensus exists on the best treatment, and thus is the treatment decision made based on both patient and lesion characteristics).^9,18,25^ Lesions are primarily treated nonoperatively. If this fails, operative treatment is usually indicated. Small primary lesions can be treated using (arthroscopic) bone marrow stimulation techniques.^6^ In case of a fragmentary lesion, fixation is advised to be used (eg, using the Lift, Drill, Fill, and Fix technique).^17,24^ However, for larger symptomatic (non-)primary lesions, more invasive techniques are often needed such as osteochondral auto- or allograft transplantation.^16,34^

An example of osteoperiosteal transplantation is using a press-fit autograft from the ipsilateral technique, named Talar OsteoPeriostic grafting from the Iliac Crest (TOPIC). For medial osteochondral lesions of the talus, good to excellent clinical, radiologic, and safety outcomes have been observed.^10^ However, although the majority of the lesions are located on the medial side of the talar dome, 24% of OLTs are located on the lateral dome.^32^ Lesions located on the lateral talar dome are often treated substantially different to medially located lesions. Medial lesions are frequently treated using a medial malleolar osteotomy but may also be accessed through anteromedial or posteromedial approaches. Lateral lesions, however, often need an ATFL release at the fibular footprint, but an osteotomy or posterolateral approach is possible in some cases.^11^ Moreover, it has been reported that medial lesions have better outcomes compared with lateral OLTs.^2^ Therefore, the TOPIC procedure for lateral lesions may have different outcomes and other safety concerns compared with the medial TOPIC technique. To date, however, no reports have been published.

It is therefore the primary aim of the present prospective study to investigate the clinical outcome, measured by the numeric rating scale (NRS) for pain during walking, at 2-year follow-up after the TOPIC procedure for lateral OLTs. The secondary aim is to assess other clinical, radiologic, and safety outcomes at both 1- and 2-year follow-up.

## Materials and Methods

The present study is a single-center, nonrandomized cohort study. It was approved by the Medical Ethical Committee of the Amsterdam UMC, location AMC, University of Amsterdam with reference number MEC 08/326. Moreover, the study has been conducted in accordance with the Declaration of Helsinki and the Dutch Medical Research Involving Human Subjects Act (WMO).

### Patient Selection

All patients who underwent a press-fit TOPIC procedure for an OLT of the lateral talar dome in the Amsterdam UMC were eligible for inclusion in the present study. The inclusion and exclusion criteria are outlined in Table 1. The indication of the lateral TOPIC technique is a symptomatic (ie, deep ankle pain during activity), large (>10 mm) OLT that does not respond to nonoperative treatment of at least 3-6 months.^11,25^ Both primary and nonprimary lesions were included.

**Table 1.** Inclusion and Exclusion Criteria.

Inclusion	Exclusion
OLT of the lateral talar dome treated with a press-fit TOPIC graft	<2 y of follow-up
	Concomitant osteochondral lesion of the tibial plafond (OLTP)
	Comorbidities (eg, malignancy, active infectious ankle pathology, hemophilic or diffuse arthropathy)
	Advanced ankle osteoarthritis (tibiotalar osteoarthritis of grade III or IV, as described by Kellgren and Lawrence)

Abbreviations: OLT, osteochondral lesions of the talus; TOPIC, Talar OsteoPeriostic grafting from the Iliac Crest.

### Treatment

All patients were treated by an experienced fellowship-trained foot and ankle academic orthopaedic surgeon (G.K. or S.A.S.). The surgical technique including aftertreatment has been previously described.^11^

In summary, an anterolateral approach is used in which the ATFL is released from the footprint at the fibula, thereby allowing exposure of the OLT on the lateral talar dome. The lesion is excised in a rectangular shape, after which the base of the defect is debrided, drilled, and measured. An osteoperiosteal autograft is harvested from the iliac crest, which is 1 mm larger in all dimensions than the excised lesion (anteroposterior, mediolateral, and depth). This graft is then positioned 1 to 2 mm subchondral in the talar dome using a press-fit technique. Thereafter the ATFL is reinserted, and in case of gross chronic instability and nonviable ligamentous structures augmented using an InternalBrace. Lastly, the incision layers are closed. Patients are then placed in a nonweightbearing cast for 6 weeks, followed with a walking boot for another 6 weeks. This is done to ensure the incorporation of the iliac crest graft.

### Outcome Assessment

All outcomes were prospectively assessed, both preoperatively and postoperatively.

#### Clinical outcomes

Clinical outcomes were assessed using electronic questionnaires using CASTOR, both preoperatively and postoperatively at the 6-month, 1-year, and 2-year follow-up. These questionnaires consisted of the numeric rating scale (NRS) of pain during walking, during rest, and during stairclimbing. Additionally, all subscales of the Foot and Ankle Outcome Score (FAOS) and the mental and physical component summary scores of the 36-Item Short Form Health Survey (SF-36) and the American Orthopaedic Foot & Ankle Society (AOFAS) were assessed.^1,3,29^

#### Radiologic evaluation

Radiographic assessment was conducted preoperatively and 12 weeks, 1 year, and 2 year postoperatively using computed tomography (CT). All radiologic variables were measured by two researchers independently and for the categorical variables in case of inconsistencies a discussion was held. The senior author’s (S.A.S.) assessment was decisive if consensus could not be reached.

Preoperatively, the lesion surface area (maximal mediolateral diameter × maximal anteroposterior diameter × 0.79) and lesion volume (maximal mediolateral diameter × maximal anteroposterior diameter × depth) were determined.^6^ No intraclass correlations were calculated, as a previous study showed good to excellent inter- and intraobserver agreement.^10^ Additionally, lesion morphology (fragmentary, cystic, or craterlike), location (9-grid scheme), and ankle OA score were assessed.^7,23,26^ At 12 weeks, incorporation of the TOPIC graft was assessed using CT. This was also performed at 1 and 2 years of follow-up as well as the development of cysts in or around the TOPIC graft.

#### Safety outcomes

All complications were assessed, which were defined as “any undesirable, unintended and direct result of an operation affecting the patient.”^13,30^ Moreover, all reoperations (either graft- or non-graft-related) were recorded.

### Statistical Analysis

All numerical variables were assessed for normality using visual inspection of box plots and the Shapiro-Wilk test. In case of normality, means with SDs were presented. If data were non-normally distributed, medians with interquartile ranges (IQRs) were presented. Categorical variables are presented as absolute numbers with percentages. The preoperative and 2-year postoperative clinical outcomes were compared using the paired *t* test (normally distributed) or Wilcoxon signed-rank test (non-normally distributed). A significance level (α) of .05 was used.

All statistical analyses were performed in Python (version 3.11.4) using package SciPy (version 1.11.2)^33,35^

### Source of Funding

No external funding was received for this study.

## Results

### Patient Selection and Characteristics

**Figure 1.**
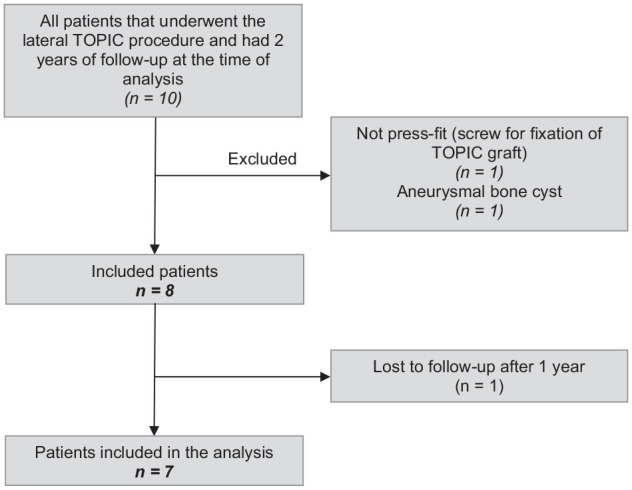
Patient selection flow chart.

In total, 10 patients underwent the lateral TOPIC procedure more than 2 years ago within the Amsterdam UMC. After applying the inclusion and exclusion criteria, 8 patients were eligible for inclusion in the present study. One patient was lost to follow-up after 1-year. Therefore, 7 patients were included in the analysis (Figure 1), of which 4 patients received an InternalBrace. The demographic and lesion characteristics can be appreciated in Tables 2 and 3, respectively.

**Table 2.** Patient Characteristics at Baseline.

Characteristic	Value
Age at surgery, y, median (IQR)	31.1 (24.5-33.6)
Sex, male/female, n (%)	3 (43) / 4 (57)
BMI, median (IQR)	23.1 (22.4-23.8)
Laterality, right/left, n (%)	4 (57) / 3 (43)
Smoking, yes/no, n (%)	2 (29) / 5 (71)
InternalBrace use, yes/no, n (%)	4 (57) / 3 (43)

Abbreviations: BMI, body mass index; IQR, interquartile range.

**Table 3.** Lesion Characteristics at Baseline.

Characteristic	Value
Nature, primary/nonprimary, n (%)	6 (86%) / 1 (14%)
Lesion size, median (IQR)	
AP, mm	16 (14-18)
ML, mm	10 (9-13)
Depth, mm	8 (6-11)
Surface area, mm^2^	126 (110-147)
Volume, cm^3^	1.2 (0.9-1.7)
Lesion location, n (%)	
Zone 3	2 (29)
Zone 6	4 (57)
Zone 9	1 (14)
Lesion morphology, n (%)	
Cystic	7 (100)
Preoperative ankle OA stage, n (%)	
Stage 1	4 (57)
Stage 2	3 (43)

Abbreviations: AP, anteroposterior; IQR, interquartile range; ML, mediolateral; OA, osteoarthritis

#### Clinical outcomes

##### Primary outcome

**Figure 2.**
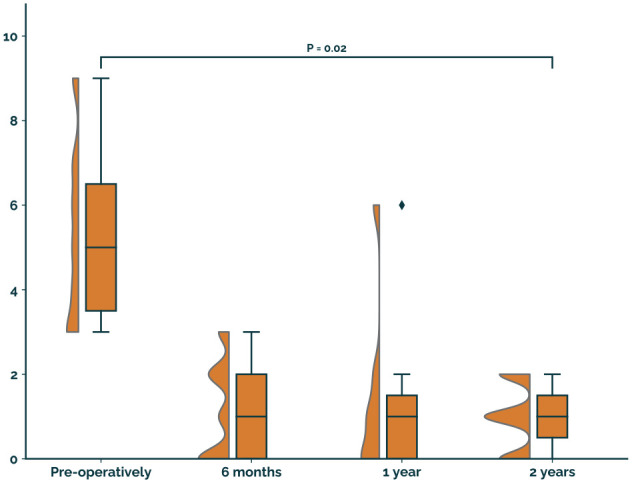
NRS of pain during walking.

The NRS of pain during walking improved from 5 (4-7) preoperatively to 1 (0-2) at 2-year follow-up (*P* = .02; Figure 2).

##### Secondary clinical outcomes

The baseline and 2-year outcomes can be appreciated in Table 4. The AOFAS improved from median 54 to 100 (*P* = .02). All FAOS subscales, except the FAOS symptoms (57 to 54, n.s.) improved significantly. Visual representation using boxplots are available in Appendix 1 until 10.

**Table 4.** Secondary Clinical Outcomes.

Outcome	Preoperative, Median (IQR)	2-y Follow-Up, Median (IQR)	*P* Value
NRS during rest	1 (0-3)	0 (0-1)	n.s.
NRS during stairclimbing	7 (6-9)	1 (0-2)	0.03
AOFAS	54 (49-59)	100 (95-100)	0.02
FAOS symptoms	57 (46-61)	54 (52-86)	n.s.
FAOS pain	58 (52-70)	92 (88-99)	0.03
FAOS ADL	69 (55-82)	97 (91-100)	0.03
FAOS sports	30 (25-38)	90 (80-98)	0.02
FAOS QoL	25 (16-31)	62 (56-85)	0.03
SF-36 PCS	36 (32-38)	36 (28-36)	n.s.
SF-36 MCS	43 (32-46)	46 (43-51)	n.s.

Abbreviations: ADL, activities of daily living; AOFAS, American Orthopaedic Foot & Ankle Society; FAOS, Foot and Ankle Outcome Score; MCS, mental component summary; NRS, numeric rating scale; PCS, physical component summary; QoL, quality of life; SF-36, 36-Item Short Form Health Survey.

##### Radiologic outcomes

All patients (100%) were available for radiologic follow-up at 3 months postoperatively. At 1 year, 1 patient was not available because of pregnancy. At 2-year follow-up, 1 was not available for radiologic follow-up.

All patients (100%) showed graft incorporation at the 3-month follow-up CT scan. At the 2-year follow-up, 5 of 6 patients showed cyst presence in or around the TOPIC graft (Table 5).

**Table 5.** Radiologic Outcomes.

Outcome	3 mo, n (%) (n = 7)	1 y, n (%) (n = 6)	2 y, n (%) (n = 6)
Graft consolidation	7 (100)	6 (100)	6 (100)
Cyst presence, yes/no	N/A	4 (67) / 2 (32)	5 (83) / 1 (17)

##### Safety outcomes

No complications occurred and none of the patients complained of donor site morbidity. None of the patients underwent any additional surgery.

## Discussion

The present study shows that the TOPIC procedure for large lateral OLTs is a promising treatment option in the first 7 patients with an improvement from preoperatively a median of 6 to a median of 1 at 2-year follow-up on the NRS of pain during walking. Moreover, no safety concerns have been observed.

The change of 4 on the NRS of pain during walking is similar to what Dahmen et al^10^ found for the medial TOPIC procedure at 2-year follow-up. No patients underwent subsequent surgery in the present study, compared with a rate of 37% of the patients undergoing the TOPIC for a medial OLT need to undergo surgery again before 2 years of follow-up. This difference in the reoperation rate is, however, mainly attributable to the removal of the hardware that is used to fixate the medial malleolar osteotomy in case of a medial TOPIC, which is not the case in the lateral procedure.

Radiologically, 100% of the grafts incorporated within 3 months and did not disintegrate in the follow-up period. In 5 of 6 patients, cysts were present in or around the TOPIC graft at 2-year follow-up. This rate of cyst formation is in line with the existing literature on osteochondral and osteoperiosteal autografting.^14,19,20,31,36,37^ Of these, 2 studies^31,37^ assessed a correlation with visual analog scale pain score and 3 studies^31,36,37^ with the AOFAS outcome score. None of these studies have found any correlation. Moreover, in the 2-year outcomes of the medial TOPIC procedure, no relation has been observed between cyst presence and the NRS of pain during walking.^10^

In the literature, most studies have focused on medial OLTs. Al-Shaikh et al^2^ reported that medial lesions have better outcomes when compared to lateral lesions after osteochondral autografting. However, for bone marrow stimulation, the opposite has been described.^5^

The TOPIC procedure provides several benefits when compared to other available techniques for large (cystic) lateral OLTs. One of the advantages is the limited costs of the TOPIC procedure when compared to techniques such as osteochondral allograft transplantation or (2-stage) cartilage implantation techniques. Moreover, the osteoperiosteal graft from the iliac crest mirrors the curvature of the talar dome, and because of the periosteal layer, it also may have chondrogenic potential.^15,21,22^ Moreover, donor site morbidity is less frequently observed when compared to osteochondral autograft transplantation techniques in which the ipsilateral knee is used.^27,28^ When performing the TOPIC procedure for a lateral OLT, the graft should be placed 1 to 2 mm subchondrally. Proudness of the graft might induce chondral damage to the opposing tibial plafond. However, the final thickness of the periosteal layer could not be measured in the present study.

The main strengths of the present study are that it is a prospective study with 83% radiologic follow-up. Additionally, all radiologic outcomes were extracted and cross-verified by 2 researchers independently from each other. The main limitation of the present study is however the limited sample size and thus low statistical power, which made it impossible to do any subgroup analyses. Moreover, the surgeries were performed by a highly experienced team that may not be generalizable to other settings. The results provided in this article for the clinical success need to be confirmed in different settings, with larger cohorts and optimally with comparative studies.

The clinical relevance of the present study is that it is the first to report on the outcomes after osteoperiosteal autografting from the iliac crest for specifically lateral OLTs. Cao et al^4^ and Guo et al^12^ have respectively included 9 and 3 lateral patients but did not analyze these separately from medial lesions. Overall, these studies showed effective outcomes. Based on the results in the present study, further research is justified on this novel technique for lateral OLTs. The effect sizes and variances reported here can be used to power future studies.

## Conclusion

In the first 7 prospectively followed patients who underwent the TOPIC procedure for large osteochondral lesions of the lateral talar dome, an improvement of the NRS of pain during walking from preoperatively (median 5) to 2-year follow-up (median 1) was observed. Additionally, all grafts showed incorporation, and no reoperations and complications were recorded. Cysts developed in up to 5 of 6 patients. The results of this study warrant further statistically powered research, including a higher number of patients and long-term outcomes.

## Supplemental Material

sj-docx-2-fai-10.1177_10711007251329033 – Supplemental material for Talar OsteoPeriostic Grafting From the Iliac Crest (TOPIC): Prospective 2-Year Outcomes for Large Lateral Osteochondral Lesions of the Talus Supplemental material, sj-docx-2-fai-10.1177_10711007251329033 for Talar OsteoPeriostic Grafting From the Iliac Crest (TOPIC): Prospective 2-Year Outcomes for Large Lateral Osteochondral Lesions of the Talus by Julian J. Hollander, Kaj S. Emanuel, Jari Dahmen, Gino M. M. J. Kerkhoffs and Sjoerd A. S. Stufkens in Foot & Ankle International

sj-pdf-1-fai-10.1177_10711007251329033 – Supplemental material for Talar OsteoPeriostic Grafting From the Iliac Crest (TOPIC): Prospective 2-Year Outcomes for Large Lateral Osteochondral Lesions of the Talus Supplemental material, sj-pdf-1-fai-10.1177_10711007251329033 for Talar OsteoPeriostic Grafting From the Iliac Crest (TOPIC): Prospective 2-Year Outcomes for Large Lateral Osteochondral Lesions of the Talus by Julian J. Hollander, Kaj S. Emanuel, Jari Dahmen, Gino M. M. J. Kerkhoffs and Sjoerd A. S. Stufkens in Foot & Ankle International
