# Supplementary material for: Talar OsteoPeriostic Grafting From the Iliac Crest (TOPIC): Prospective 2-Year Outcomes for Large Lateral Osteochondral Lesions of the Talus
Source: Foot Ankle Int. 2025 Apr 27;46(6):580–6. doi: 10.1177/10711007251329033 (PMC12145484; doi:10.1177/10711007251329033)
Supplement: sj-docx-2-fai-10.1177_10711007251329033 – Supplemental material for Talar OsteoPeriostic Grafting From the Iliac Crest (TOPIC): Prospective 2-Year Outcomes for Large Lateral Osteochondral Lesions of the Talus [file sj-docx-2-fai-10.1177_10711007251329033.docx]

**Appendix 1: NRS during Rest**


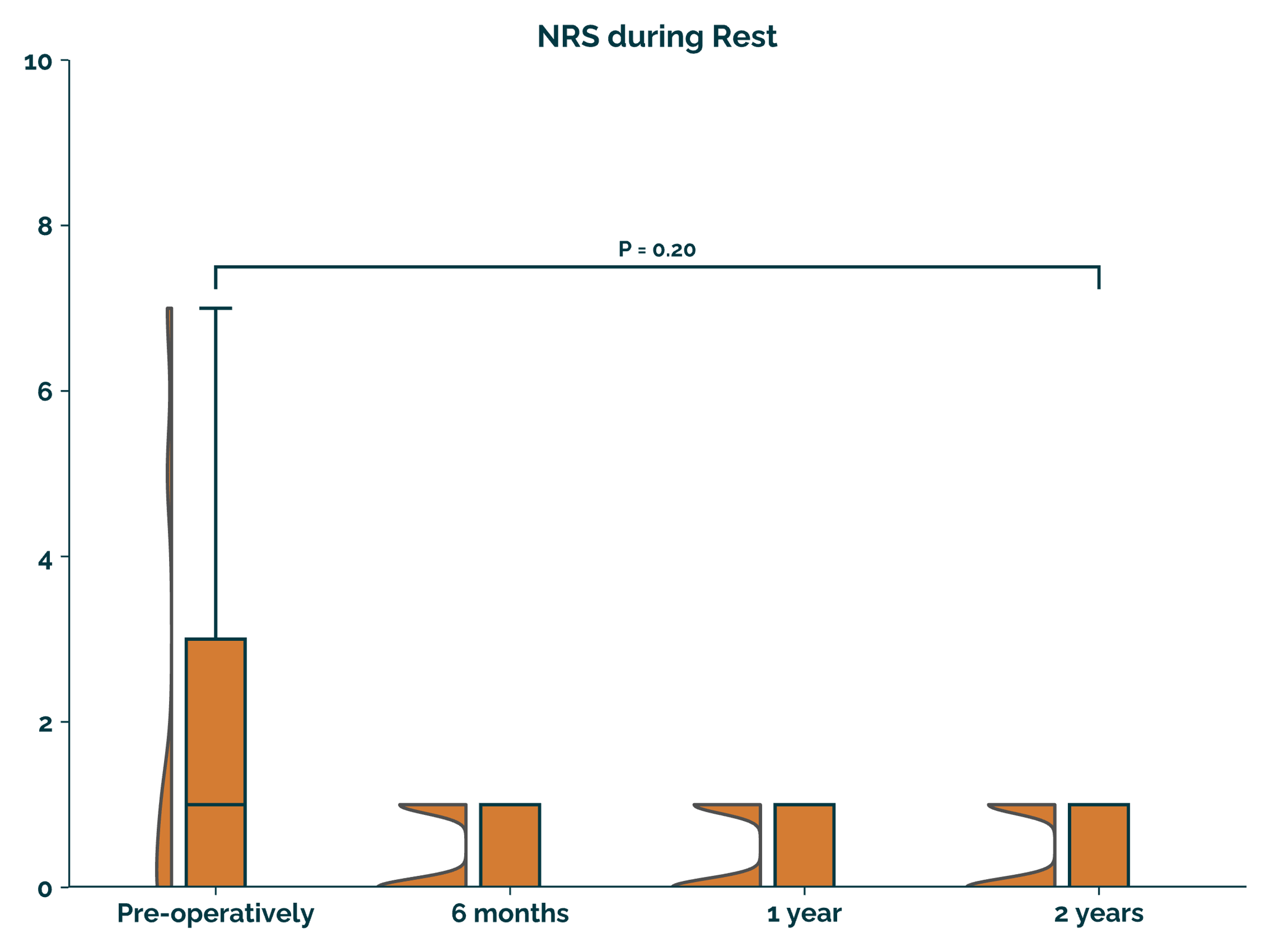


**Appendix 2: NRS during Stairclimbing**


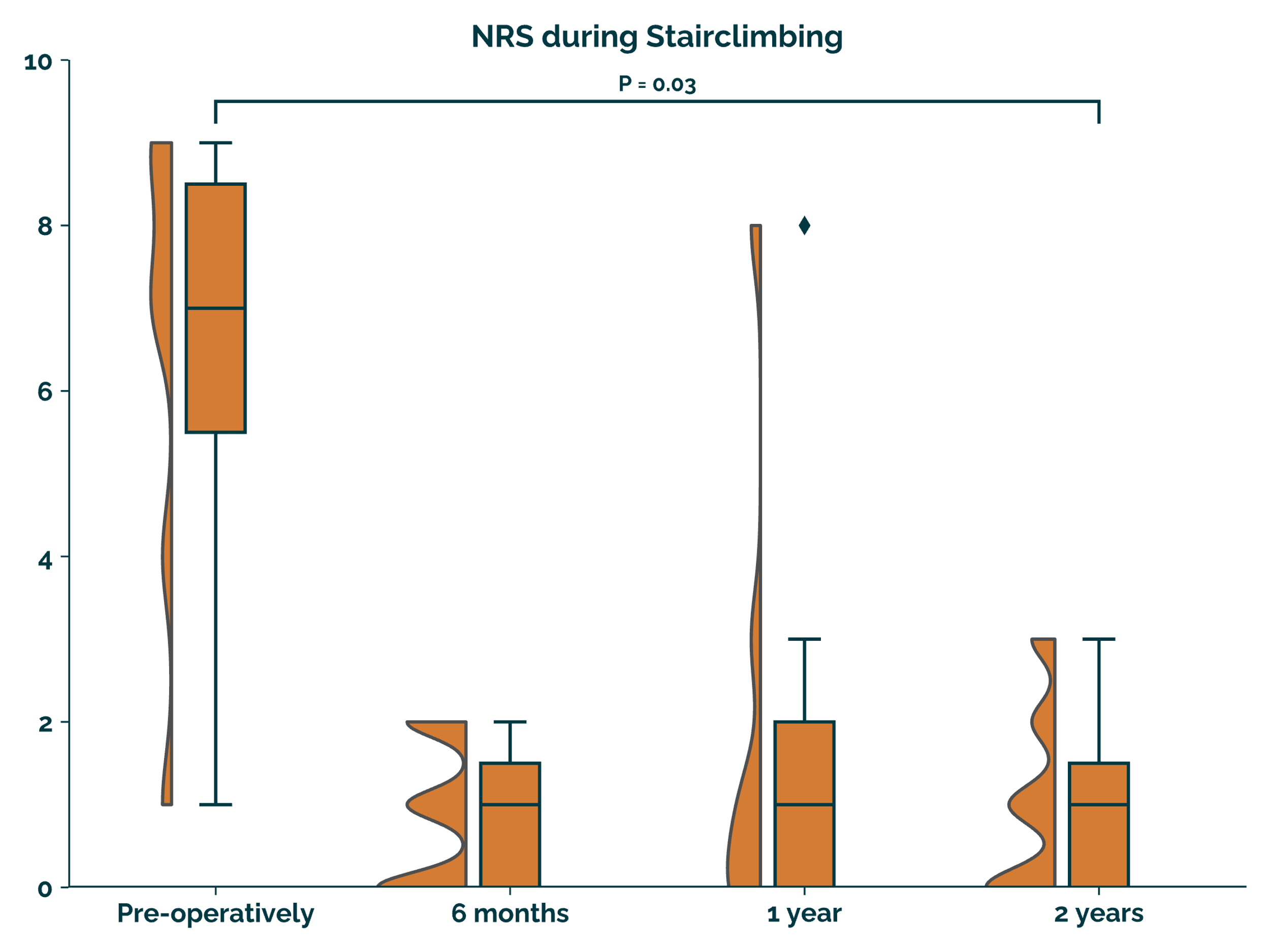


**Appendix 3: AOFAS**


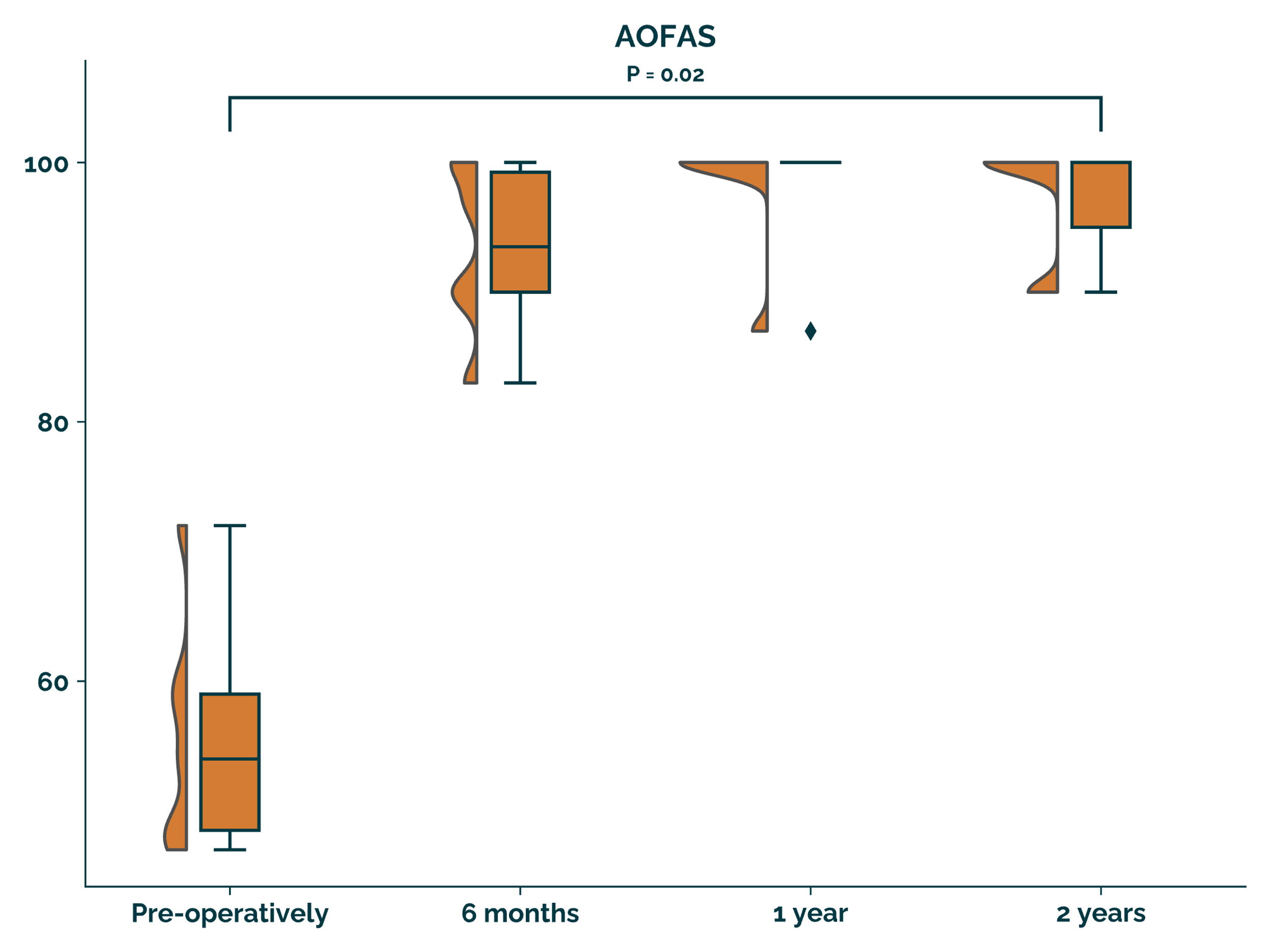


**Appendix 4: FAOS Symptoms**


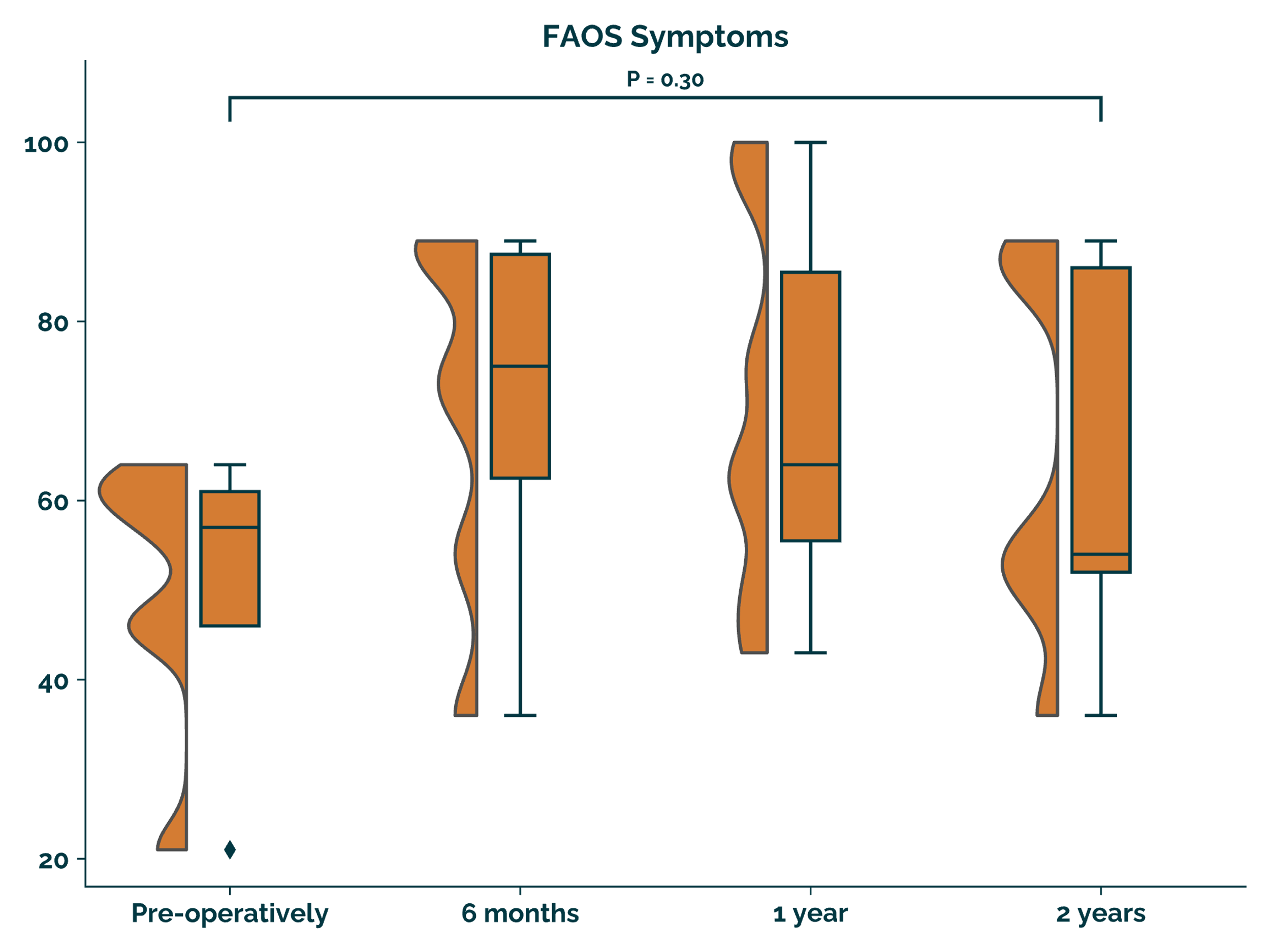


**Appendix 5: FAOS Pain**

**
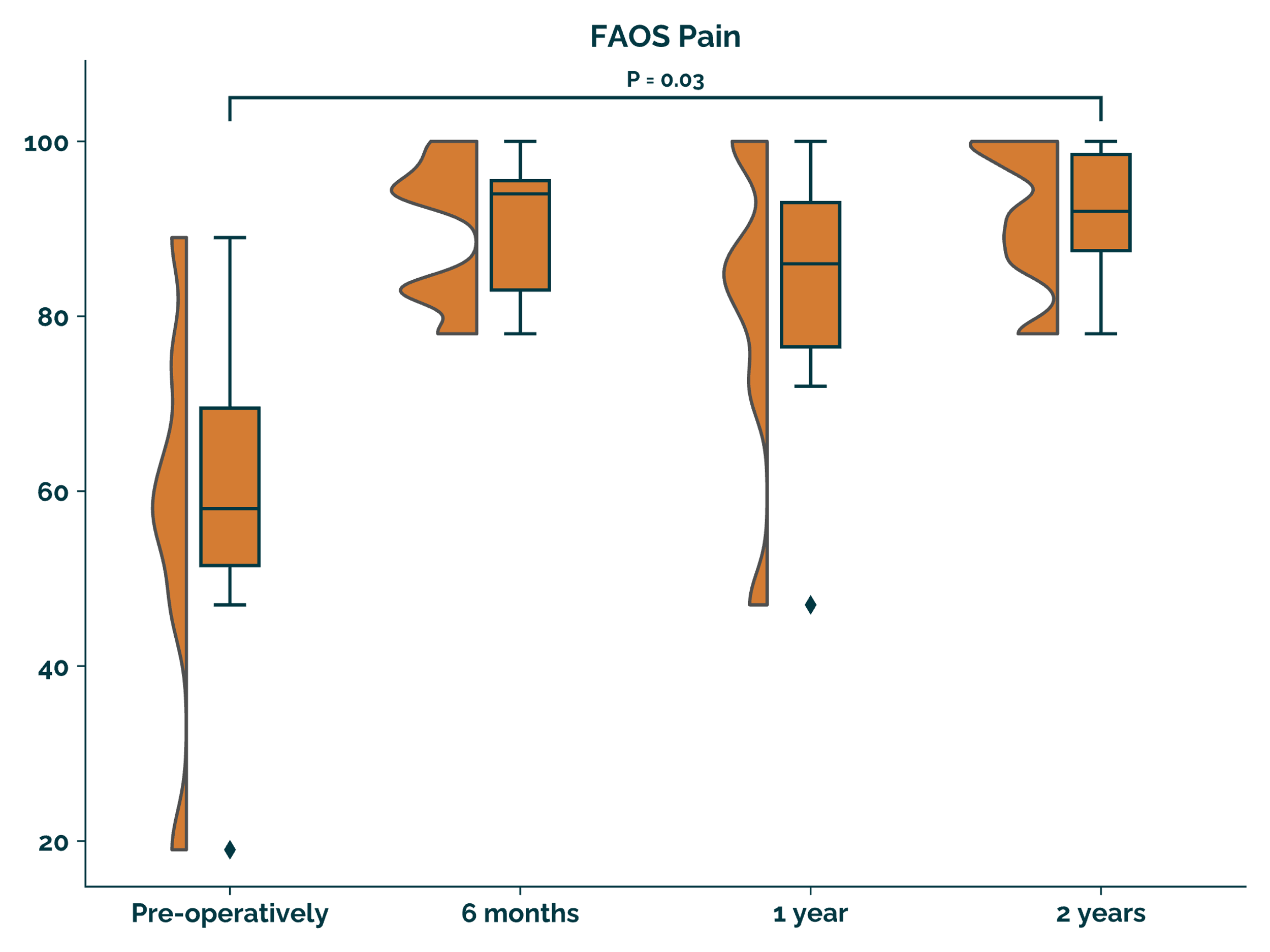
**

**Appendix 6: FAOS ADL**

**
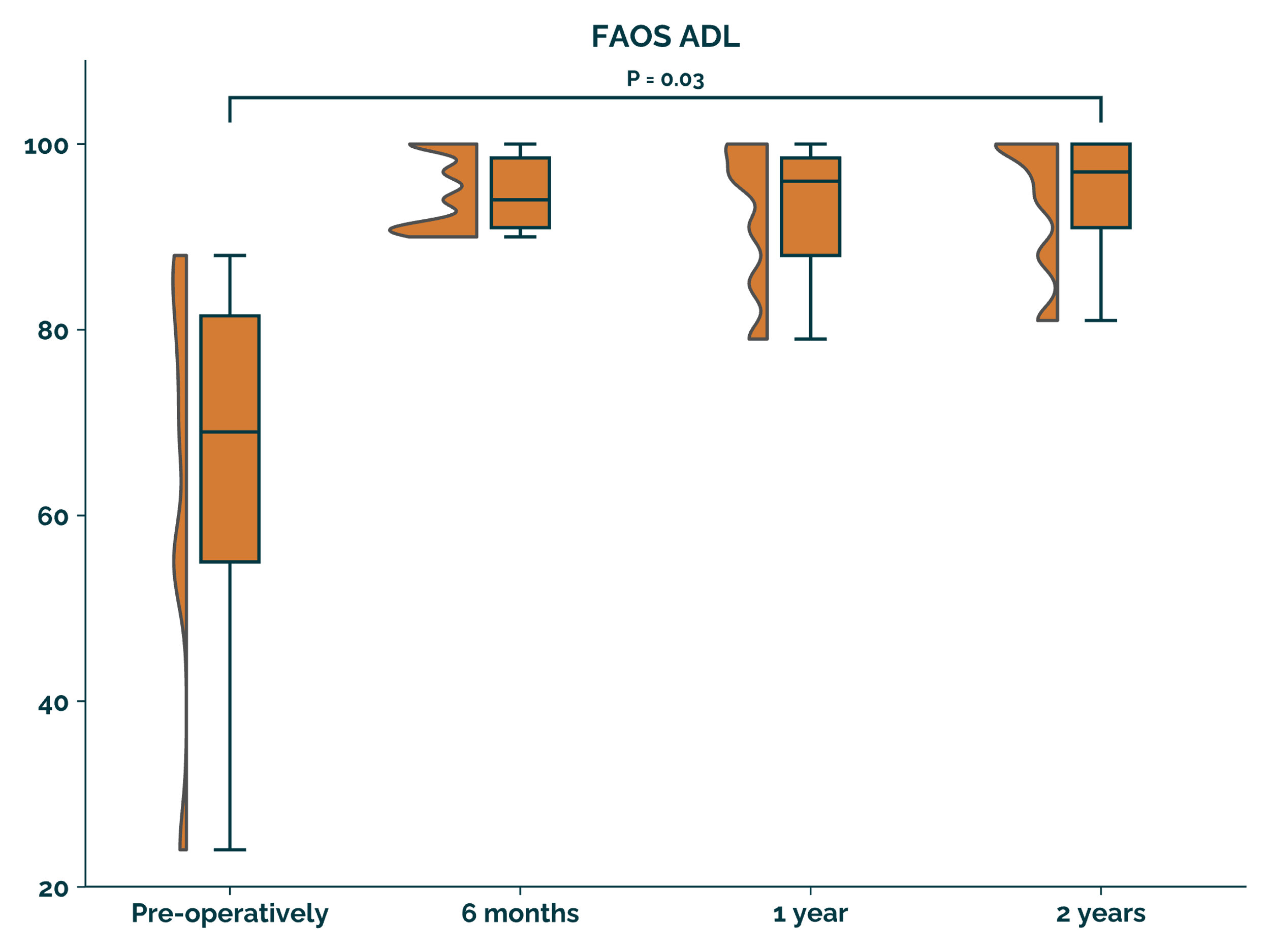
**

**Appendix 7: FAOS Sports**

**
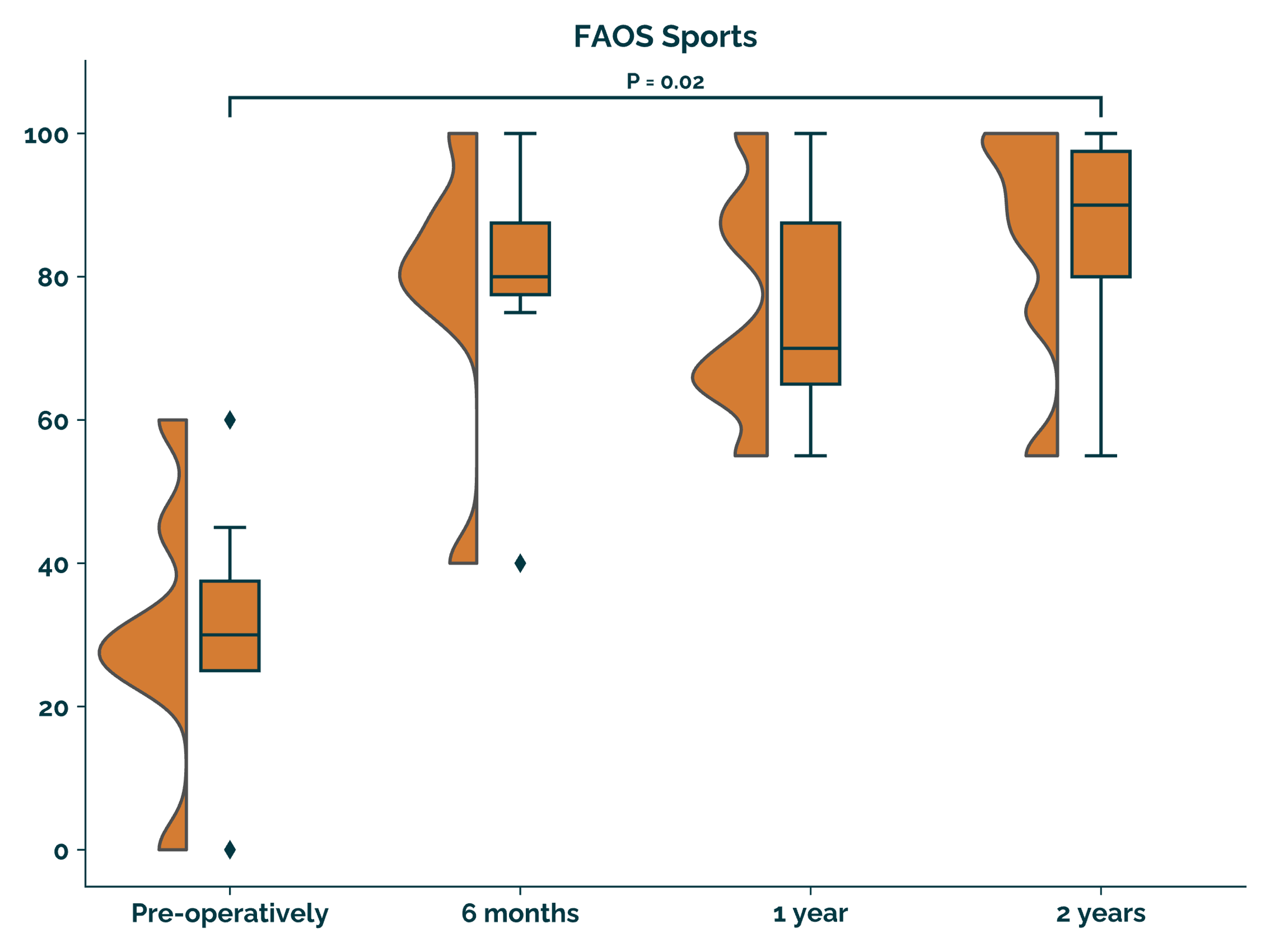
**

**Appendix 8: FAOS QoL**

**
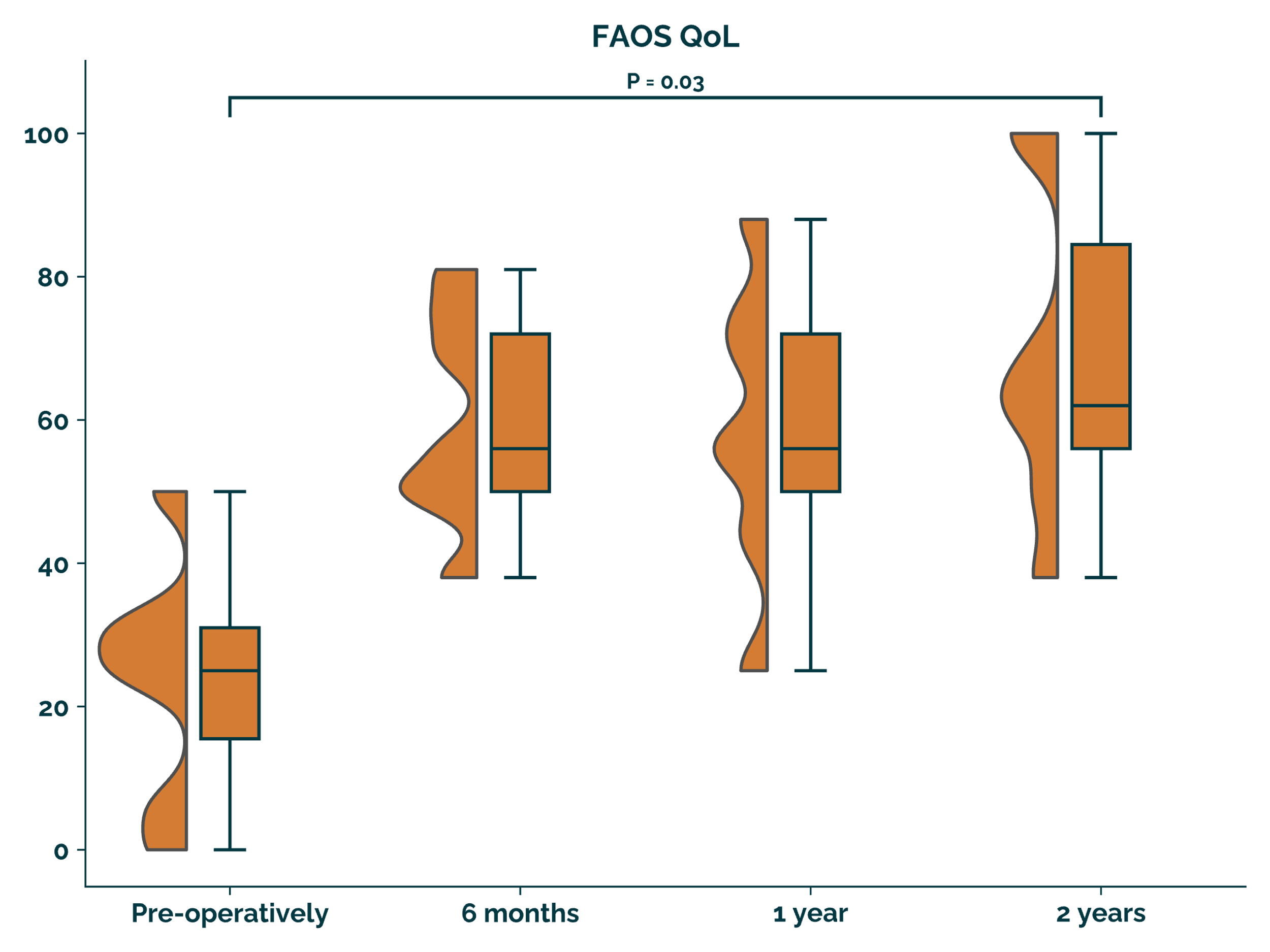
**

**Appendix 9: SF-36 PCS**

**
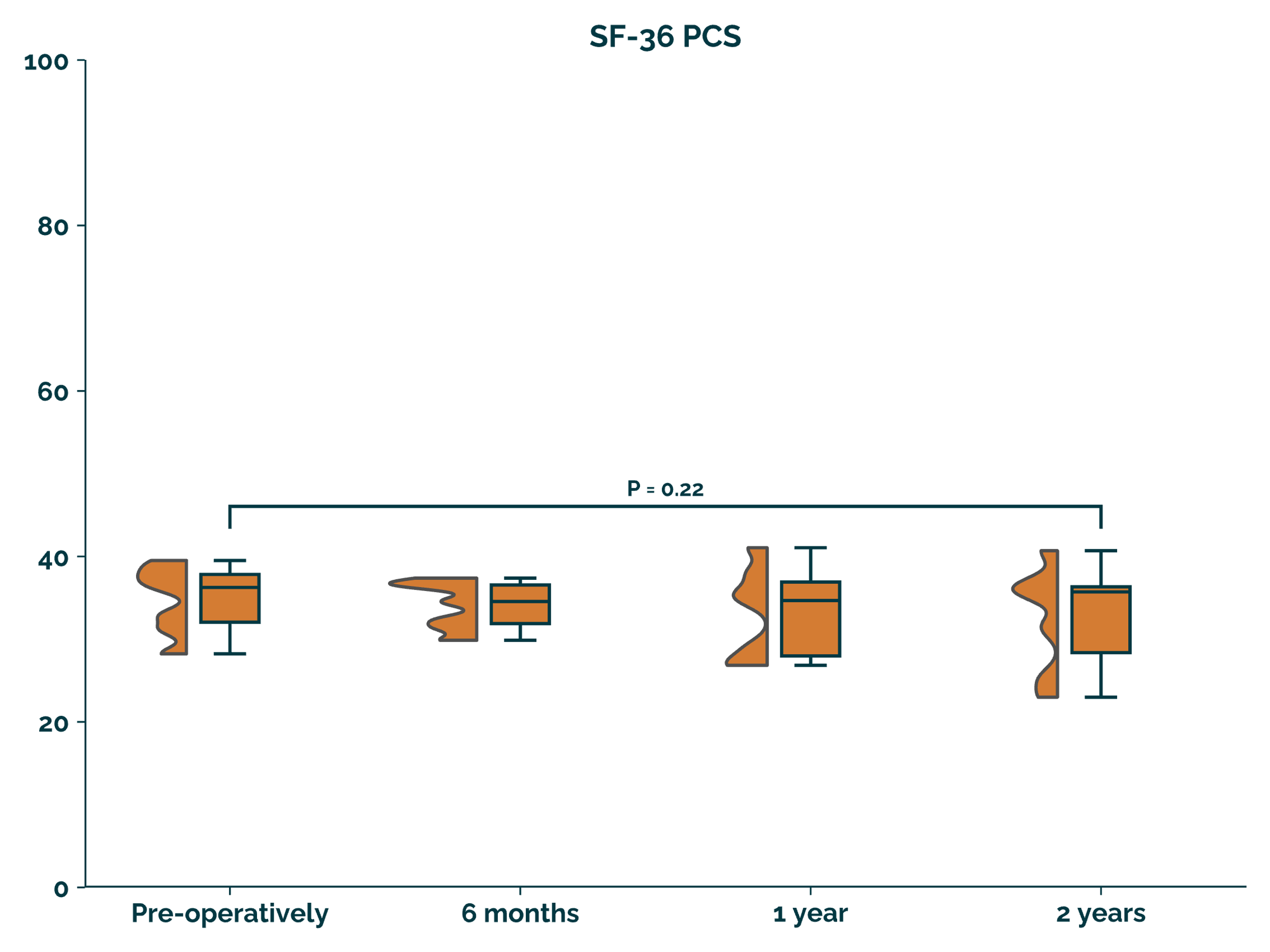
**

**Appendix 10: SF-36 MCS**

**
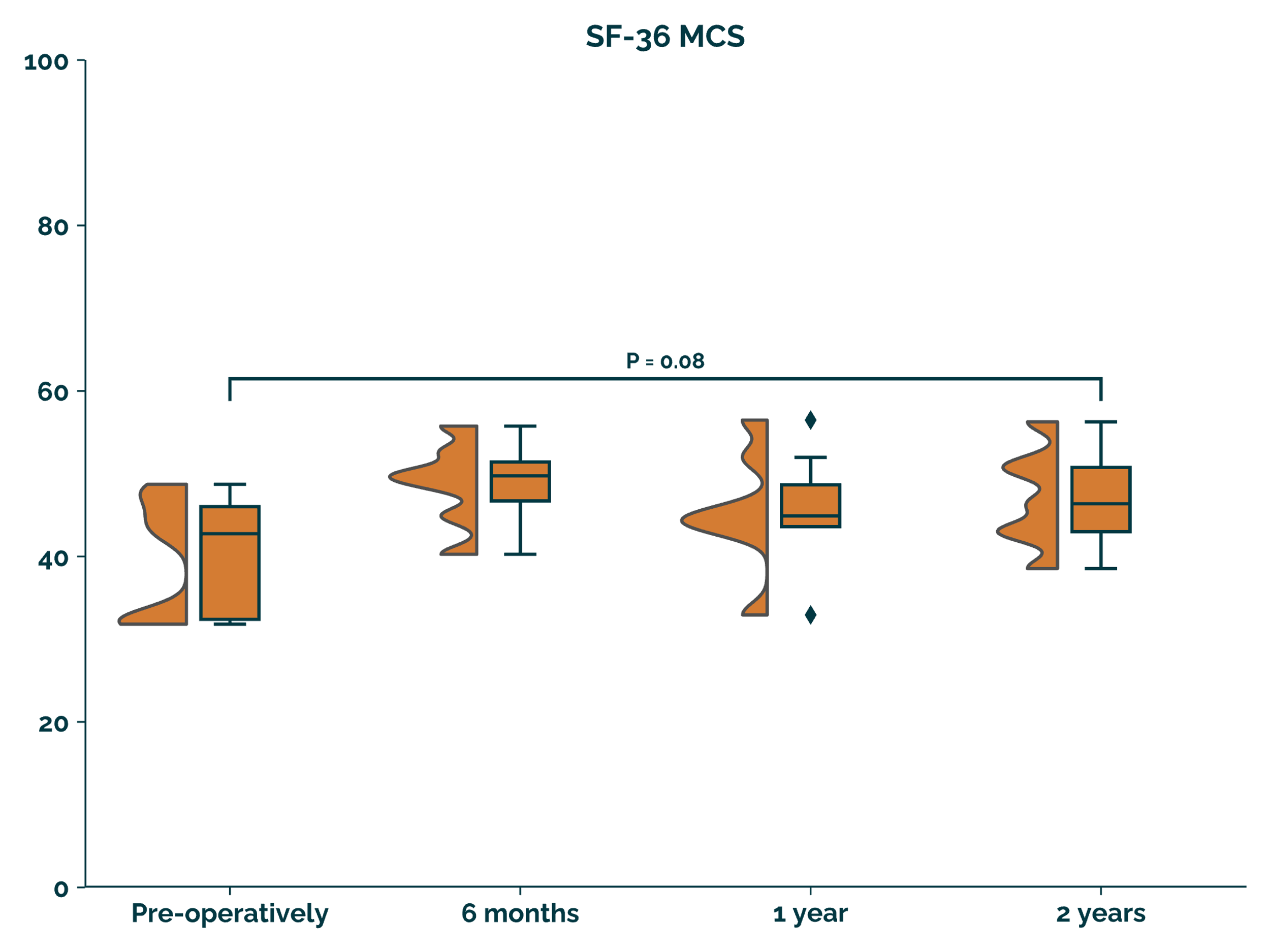
**
